# Effects of feeding hybrid rye silage as a replacement for barley silage on feed intake, ruminal fermentation, and the site and extent of nutrient digestion in growing beef heifers

**DOI:** 10.1093/jas/skaf230

**Published:** 2025-07-22

**Authors:** Fuquan Zhang, Rebecca S Brattain, Herman Wehrle, Vern S Baron, Gabriel O Ribeiro, Gregory B Penner

**Affiliations:** Department of Animal and Poultry Science, University of Saskatchewan, Saskatoon, SK, Canada S7N 5A8; KWS Cereals USA, LLC, Champaign, IL, USA 61822; KWS Seeds Canada, Calgary, AB, Canada T2T 0A4; Agriculture and Agri-Food Canada, Lacombe Research and Development Centre, Lacombe, AB, Canada T4L 1W1; Department of Animal and Poultry Science, University of Saskatchewan, Saskatoon, SK, Canada S7N 5A8; Department of Animal and Poultry Science, University of Saskatchewan, Saskatoon, SK, Canada S7N 5A8

**Keywords:** silage, growing cattle, hybrid rye, ruminal fermentation

## Abstract

The objective of this study was to evaluate the effects of feeding hybrid rye silage (HRS) as a replacement for barley silage (BARS) on dry matter intake (DMI), ruminal fermentation, and the site and extent of digestion when fed to growing beef heifers. Eight ruminally cannulated Hereford × Simmental heifers (519 ± 25.8 kg BW) were used in a replicated 4 × 4 Latin square design with 28-d periods, including 21 d for dietary adaptation and 7 d for data and sample collection. Treatments included a control diet (BCON) that contained 59.62% BARS (harvested at the soft dough stage of maturity), 38.61% barley grain, and 1.78% of a vitamin and mineral supplement on a dry matter (DM) basis. The HRS (harvested at the boot stage of maturity) replaced 33% (RLOW), 67% (RMED), or 100% (RHIGH) of the BARS on a DM basis. Increasing HRS inclusion linearly (*P* = 0.04) decreased DMI, linearly increased water intake (*P* = 0.02) and mean ruminal pH (*P* < 0.01) and linearly decreased the duration that ruminal pH was < 5.8 (*P *= 0.04). There was no effect of HRS inclusion on total short-chain fatty acid (SCFA) concentration (*P* = 0.46) in ruminal fluid, but the molar proportion of acetate linearly (*P* < 0.01) increased while propionate and butyrate linearly decreased (*P* < 0.01). The NH_3_-N concentration linearly increased (*P* = 0.03) with increasing HRS inclusion. Increasing HRS inclusion increased ruminal degradability of DM, OM, and aNDFom, while there was no effect on intestinal digestibility of nutrients, either expressed as a percent of intake or percent of digesta flow. Total tract digestibility of DM (*P* ≤ 0.01), ADF (*P* ≤ 0.01), and aNDFom (*P* ≤ 0.01) increased with increasing HRS inclusion, but inclusion did not affect gross energy (GE) digestibility (*P* = 0.15) and the dietary digestible energy (DE) concentration (*P* = 0.17). In conclusion, the use of HRS, harvested at the boot stage, as a direct replacement for BARS, harvested at the soft dough stage, is likely to reduce DMI but improve NDF digestibility without affecting dietary DE concentration. As there was no difference in GE digestibility or dietary DE digestibility, these results demonstrate a tradeoff between greater NDF digestibility of HRS compared to BARS but reduced DMI with increasing inclusion.

## Teaser

Replacing barley silage with hybrid rye silage in diets for growing beef cattle decreased DMI but increased ruminal OM and NDF digestion, leading to no difference in the dietary digestible energy concentration.

## Introduction

Whole-crop barley (*Hordeum vulgare L*.) silage (**BARS**) is commonly used for feedlot cattle in Western Canada (Nair et al., 2016; Johnson et al., 2020). While barley has been a staple forage source, the development of new varieties of hybrid fall rye (*Secale cereale L*.) allow for fall seeding and earlier silage harvest than barley (KWS Production Guide Hybrid Rye, 2023), thereby spreading production risk and capitalizing on fall and early spring moisture. Growing fall rye for silage has been reported to produce similar yield when compared to spring-seeded barley (Zhang et al., 2025), or even greater yield for hybrid rye relative to other small cereal grain forages seeded in fall and early spring (Geiger and Miedaner, 2009; KWS Production Guide Hybrid Rye, 2023). Equal or greater yield for hybrid rye relative to spring-seeded barley, along with the earlier harvest date, may allow for a second crop to be seeded in the same growing season (Darambazar et al., 2022). While production of rye silage has been adopted, few studies have evaluated the use of rye harvested as silage (**HRS**) for feedlot cattle (Stefanyshyn-Cote, 1993; Zhang et al., 2025).

Hybrid rye silage harvested at the late milk stage of maturity was reported to have greater concentrations of acid detergent fiber (ADF) and neutral detergent fibre (NDF), but less starch than BARS harvested at the soft dough stage of maturity (Zhang et al., 2025). Previous studies have characterized how the physiological growth stage for small grain species (barley, oats, rye, wheat, and triticale) harvested as forage affects dry matter (DM) yield, morphological and anatomical characteristics, and forage quality (Edmisten et al., 1998a, 1998b; Sartin et al., 2022). Results from those studies indicate that when harvested at the same maturity, the in vitro dry matter disappearance (IVDMD) of cereal rye silage was generally less than that of barley, oat, and wheat throughout the milk to hard dough stages (Edmisten et al., 1998b). Moreover, fall rye silage harvested either at flowering or at mid-milk resulted in lesser DMI for growing steers, and lesser in vivo digestibility of most nutrients at the mid-milk stage when compared to barley silage harvested at mid-dough (Stefanyshyn-Cote, 1993). However, we are unaware of in vivo studies that evaluated the site and extent of nutrient digestibility for hybrid rye silage.

Given that previous studies have suggested rye silage reduces DMI (Fisher and Lessard, 1987; Stefanyshyn-Cote, 1993; Terler et al., 2025) including a recent study where HRS replaced barley silage in diets for growing beef cattle (Zhang et al., 2025), it was hypothesized that increasing dietary HRS as a replacement for BARS would decrease DMI, increase ruminal pH, reduce the total ruminal short-chain fatty acid (SCFA) concentration, and alter the site of digestion due to increased concentrations of NDF and decreased starch in HRS relative to BARS. The objective of this study was to evaluate the effects of feeding HRS as a replacement for BARS on DMI, ruminal fermentation, and the site and extent of nutrient digestion when fed to growing beef heifers.

## Materials and Methods

The use of animals and experimental procedures was approved by the University of Saskatchewan Animal Research Ethics Board (protocol 20210069) according to the guidelines of the Canadian Council of Animal Care (Ottawa, ON, Canada).

### Animal management, experimental design, and dietary treatments

Eight Hereford × Simmental-cross heifers that were previously fit with a 10-cm cannula (model 9C; Bar Diamond Inc., Parma, ID, USA) and had an initial body weight (BW) of 519 ± 25.8 kg were housed at the University of Saskatchewan Livestock Research Barn (Saskatoon, SK, Canada). Heifers were ovariectomized at the time of ruminal cannulation and were approximately 8 mo old at the time of surgery. Heifers were housed in individual pens (9 m^2^) with ad libitum access to water and rubber mats on the floor throughout the study. Pens were scraped and washed daily at 0730 hours to remove manure. Heifers were permitted 2 h/d of exercise in an outdoor pen during pen cleaning and diet preparation, except during sample collections.

Heifers were stratified by BW into 1 of the 2 squares, and within the square, randomly assigned to 1 of the 4 treatment sequences in a replicated 4 × 4 Latin square design balanced for carry-over effects. Each experimental period lasted 28 d, including 21 d for adaptation and 7 d for sampling and data collection. Dietary exposure occurred abruptly at the start of each period. The control diet (BCON) contained (DM basis) 59.62% BARS in combination with 38.61% dry-rolled barley grain, and 1.78% of a vitamin and mineral premix. The HRS silage was included (DM basis) at 19.67% (RLOW), 39.94% (RMED), and 59.62% (RHIGH) by replacing 33%, 67%, or 100% of the BARS. The BARS in this study were harvested at the soft dough stage of maturity and the HRS was harvested at the boot stage. Diets were formulated to meet or exceed nutrient requirements of the heifers with a target weight gain of 1.3 kg/d according to the NASEM (2016) based on initial analysis of the feed ingredients collected prior to the start of the study. All diets included monensin (Rumensin premix; Elanco Animal Health, Ontario, Canada) at a targeted concentration of 33 mg/kg DM, which was consistent with Zhang et al. (2025). Dry rolled barley grain was purchased commercially from a single supplier and rolled to a processing index of 70% (Yang et al., 2000). Silages were sampled twice weekly, and the remaining ingredients were sampled once weekly to determine the DM concentrations. The DM coefficients for individual ingredients were adjusted on a weekly basis when the 3-wk running average differed by more than 2 percentage units.

Heifers were weighed on 2 consecutive days at 0900 hours (before feeding) at the start and end of each period. Total mixed rations (TMR) were prepared by hand mixing and the TMR was offered once daily at 0930 hours to ensure at least 5% refusals. The amount of feed offered and refused were recorded daily, and data from days 25 to 28 of each period were used to determine DMI based on the difference between the quantity of DM offered and refused. Water intake was measured using inline water meters (Model DLJSJ75, Daniel L. Herman Co., Hackensack, NJ, USA) for each heifer with meter readings recorded daily at 0700 hours from day 25 of each period to day 1 of the following period. Water flow through the meters was assumed to be equal to water intake (Penner et al., 2020). Ingredient samples (500 g) were collected daily from days 25 to 28, and refusal samples were collected from days 26 to day 1 of the following period and composited for each heifer proportionally to the amount refused. The ingredient and refusal samples were dried in a forced-air oven at 55 °C until achieving a constant weight to determine DM concentration. Subsequently, samples were ground to pass through a 1-mm sieve using a hammer mill (Christie-Norris Laboratory Mill, Christie-Norris Ltd, Chelmsford, UK) and sent to Cumberland Valley Analytical Service (Waynesboro, PA, USA) for chemical analysis including: analytical DM; organic matter (OM); crude protein (CP); NDF; NDF determined using sodium sulfate and α-amylase and then corrected for ash content (aNDFom); ADF; starch; ether extract; calcium; and phosphorus as described by Zhang et al. (2024).

### Particle distribution and sorting index

The particle size distribution of feed ingredients and refusals collected from days 25 to 28 of each period was determined using the Penn State Particle Separator (Nasco, Fort Atkinson, Wisconsin, USA) with aperture sizes of 19, 8, and 4 mm, and a bottom pan according to Heinrichs (2013). The sorting index was calculated according to Leonardi and Armentano (2003). Briefly, the actual intake of each Penn State Particle Separator fraction was expressed as a percentage of the predicted intake of that fraction, as determined if no sorting occurred. A lack of sorting is indicated by a sorting index value of 100%, sorting against is indicated by a value of <100%, and sorting for particles is indicated by a value of >100%.

### Ruminal fermentation

An indwelling ruminal pH measurement system (Dascor, Escondido, CA, USA) was placed in the ventral sac of the rumen in each heifer at 1900 hours on day 21 and removed after 0100 hours on day 1 of the following period to ensure completion of sample collection (Penner et al., 2006). Prior to insertion and after removal from the rumen, the pH systems were standardized in pH buffers 7 and 4 at 39 °C to characterize the linear relationship between mV and pH. The pH meters were programmed to measure and record every 5 min over the collection duration. The raw data obtained were converted from mV to pH using the beginning and ending standardizations derived from the linear regression between the pH buffer and the observed mV value, along with the assumption of linear drift between the starting and ending standardizations (Penner et al., 2006). Data were then restricted to 96 h of continuous pH measurement starting at 0700 hours on day 22 and ending at 0655 hours on day 26. Ruminal pH data were used to determine the daily average minimum, mean, and maximum pH values, and the duration and area that ruminal pH was <5.8.

Ruminal digesta was manually collected every 12 h with a 15-h offset between samples collected on consecutive days. Samples were collected at 0200 and 1400 hours on day 25, 0500 and 1700 hours on day 26, 0800 and 2000 hours on day 27, and 1100 and 2300 hours on day 28. At each timepoint, three spot samples (250 mL/region) of mixed ruminal digesta were collected from the cranial central, central, and caudal central regions, respectively. Digesta from the three regions were pooled and strained through 2 layers of cheesecloth. Subsequently, 10 mL of strained ruminal fluid was transferred into two 15-mL tubes, with one containing 2 mL of metaphosphoric acid (25% w/v) and the other containing 2 mL of sulfuric acid (1%). After gently and repeatedly inverting and mixing, an equal volume from each sample was used to create a composite sample for each heifer. Samples were stored frozen at −20 °C until analysis for SCFA (Khorasani et al., 1996) and ammonia-N concentrations (Broderick and Kang, 1980).

### Diet digestibility, site, and extent of digestion

Heifers were tethered during the collection period to facilitate ruminal infusion starting at 0900 hours on day 22 and the collection of omasal digesta and feces starting at 0200 hours on day 25. To measure omasal flow and calculate the site and extent of nutrient disappearance, Cr-ethylenediaminetetraacetic acid (Cr-EDTA; Udén et al., 1980) and ytterbium chloride (YbCl_3_; Siddons et al., 1985) were used as digesta markers. Markers were infused into the rumen using a peristaltic pump (Model 205U; Watson and Marlow, Cornwall, UK) to target at a constant infusion rate of 1 L/heifer/day, which provided 2.77 g of Cr and 2.77 g of Yb per day, respectively. Infusions were initiated on day 22 at 0900 hours through day 28 until finishing the collection. A 500-mL priming dose of each marker solution was infused on the first day of marker infusion. The marker solutions were weighed daily at 0900 hours to determine the actual infusion rate and a sample of each marker solution was collected prior to the initiation of ruminal infusions during each period for determination of Cr, and Yb concentrations.

Omasal digesta samples and representative feces samples were collected at the same time points as for ruminal digesta collection. At each sampling time point, 500 mL of omasal digesta was collected as described by Chibisa et al. (2012). Digesta samples collected within a period were pooled for each heifer into a common pail and stored frozen at −20 °C for further analysis. The composited omasal digesta samples (4 L for each heifer per period) were thawed at room temperature and then separated into the large particle, small particle, and fluid phases according to Brito et al. (2009). Each phase was then oven-dried at 55 °C until reaching a constant weight and ground to pass through a 1-mm screen (Retsch ZM 200; Haan, Germany). Additionally, 200 g of feces was collected from the rectum of each heifer at each collection time point. Samples were placed in a common pail for each heifer and stored at −20 °C until the end of the sampling period. Samples of feces were thawed and dried at 55 °C in a forced-air oven until reaching a constant weight. Feces were routinely mixed during drying to facilitate even drying and prevent mold growth. Once dried, all fecal samples were ground to pass through a 1-mm screen using a hammer mill (Christie-Norris Laboratory Mill; Christie-Norris Ltd, Chelmsford, UK) and sent to Cumberland Valley Analytical Service (Waynesboro, PA, USA) for chemical analysis as described above. The gross energy (GE) of all feed ingredients, refusals, and fecal samples were measured using bomb calorimetry (6,400 Automatic Isoperibol Calorimeter, Parr Instruments Company, Moline, IL, USA) at the University of Saskatchewan (Saskatoon, SK, Canada). Then GE digestibility and diet digestible energy (DE) concentration were determined as (GE intake—GE excreted in feces)/GE intake × 100, and (GE intake—GE excreted in feces)/DMI, respectively (NASEM, 2016).

The dried omasal fractions and fecal samples were used to determine the concentrations of Cr and Yb according to the methods as described in Zhang et al. (2024). The concentration of 240 h undigested NDF (uNDF) was determined in the large particle phase of omasal digesta, feed ingredients, and refusals samples using the in situ nylon bag technique according to Nair et al. (2017). Briefly, 1.0 g of feed and refusals, 0.5 g of large particle phase, and 1.2 g of small particle phase were weighed in triplicate into individual 5 cm × 10 cm in situ bags (6 μm pore size; Sefar America Inc., Depew, NY, USA). The bags were assigned randomly to 1 of the 4 laundry bags, each with two 1-kg weights. The laundry bags were then randomly assigned to 1 of the 2 ruminally cannulated lactating Holstein cows and were placed in the ventral sac of the rumen. All bags were incubated for 240 h. Upon removal, all bags were washed 10 times in cold water and then dried in an air-forced oven at 55 °C for 48 h. Subsequently, all samples were analyzed for NDF concentration with the use of heat-stable alpha-amylase and sodium sulfite using the Ankom 200 Fiber Analyzer according to the manufacturer’s protocol (Ankom Technology Corp, 2017). The Cr, Yb, and uNDF concentrations were used to reconstitute the omasal fractions into a single representative omasal sample for each heifer in each period using the triple-marker method developed by France and Siddons (1986). The reconstituted omasal digesta samples and fecal samples were sent to Cumberland Valley Analytical Service (Waynesboro, PA, USA) for determination of analytical DM, OM, CP, aNDFom, ADF, and starch as described by Zhang et al. (2024). The flow of omasal digesta and chemical constituents were calculated based on the triple-maker method as described by France and Siddons (1986). Fecal DM output was determined based on the infusion of Yb and fecal Yb concentration according to (Beauchemin et al., 2001).

### Statistical analysis

The ruminal cannula for one heifer on the RLOW diet came out during collections in period 1, and these data were removed. As the heifer was considered the experimental unit, the loss of data resulted in an *n* = 7 for period 1 and *n* = 8 for each of the remaining 3 periods. Data were analyzed using the GLIMMIX procedure of SAS 9.4 (SAS Institute Inc., Cary, NC, USA) with fixed effects of dietary treatment, and random effects of period and heifer nested within square. All data and their residuals were tested for normality using the Shapiro–Wilk test and visual assessment of the distribution of the data and residuals. The denominator degrees of freedom were calculated using the Kenward–Roger option and compound symmetry was used as the covariance error structure. Least squares means were obtained using the LSMEANS statement of SAS, and preplanned contrasts were used to test whether HRS inclusion responses were linear, quadratic, or cubic effects. Data were presented as least square means and the largest SEM was reported. The difference between the means were declared significant when *P* < 0.05, and tendencies are discussed when 0.05 < *P* ≤ 0.10. Due to the absence of significant cubic effects for the variables of primary interest, the cubic contrast was removed. In addition, a two-tailed *t*-test was used to confirm whether the particle sorting index for each particle size category and each treatment differed from 100% (version 9.4, SAS Institute Inc., Cary, NC, USA). The difference was considered significant when *P* < 0.05.

## Results

### Diet composition

While statistical comparisons were not evaluated for the silages or dietary treatments, HRS had numerically greater DM, ADF, and aNDFom concentrations with numerically less starch than BARS (Table 1). The particle size distribution was similar, although HRS had a slightly greater proportion of particles retained on the 19-mm sieve and less retained on the 8-mm sieve. As this was a direct substitution study, replacing BARS with HRS increased dietary DM, ADF, and aNDFom, and decreased starch (Table 2).

**Table 1.** Chemical composition, fermentation products of barley silage and hybrid rye silage^1^

Item	Ingredient^2^	
	Barley silage	Hybrid rye silage
Chemical composition, % DM^3^		
*n*	4	4
DM, %	29.62 ± 1.066	33.42 ± 1.013
OM	91.10 ± 0.650	90.04 ± 0.736
CP	14.58 ± 0.126	15.15 ± 0.173
ADF	27.38 ± 1.406	30.10 ± 1.490
NDF	47.50 ± 1.160	52.48 ± 0.818
aNDFom	45.90 ± 1.344	49.90 ± 0.812
uNDF_240-h_	15.64 ± 0.219	14.18 ± 0.980
Lignin	4.63 ± 0.296	4.58 ± 0.277
Starch	5.33 ± 0.568	0.15 ± 0.129
Ether extract	4.09 ± 0.106	3.92 ± 0.217
Ca	0.36 ± 0.067	0.44 ± 0.050
P	0.26 ± 0.008	0.36 ± 0.010
Particle size distribution, %		
>19.0 mm	23.31 ± 4.345	26.69 ± 8.846
19.0 to 8.0 mm	65.78 ± 2.626	67.94 ± 6.507
8.0 to 4.0 mm	7.40 ± 0.864	3.15 ± 1.274
<4.0 mm	3.50 ± 1.500	2.22 ± 1.140
Silage fermentation		
*n*	2	2
pH	4.02 ± 0.02	4.33 ± 0.04
Total acid, % of DM	10.09 ± 1.35	8.50 ± 1.03
Lactic acid, % of DM	8.70 ± 1.27	6.15 ± 0.07
Acetic acid, % of DM	1.39 ± 0.08	1.22 ± 0.76
NH_3_, % of DM	1.53 ± 0.03	1.98 ± 0.33

^1^DM, dry matter; OM, organic matter; CP, crude protein; ADF, acid detergent fiber; NDF, Neutral detergent fiber; aNDFom, NDF measured using α-amylase, sodium sulfite, and corrected for ash content; uNDF, undigested NDF.

^2^Barley silage was harvested at the soft dough stage of maturity, and hybrid rye was harvested at the boot stage of maturity.

^3^Chemical composition is expressed as means ± SD.

**Table 2.** Ingredients, chemical composition, and physical analysis of the experimental diets^1^

Item	Hybrid rye silage inclusion rate			
	BCON	RLOW	RMED	RHIGH
Ingredient, % DM				
Barley silage	59.62	39.94	19.67	-
Hybrid rye silage	-	19.67	39.94	59.62
Barley grain	38.61	38.61	38.61	38.61
Vitamin/mineral premix^2^	1.78	1.78	1.78	1.78
Chemical composition^3^, % DM				
DM, %	40.55 ± 0.012	41.83 ± 0.011	43.24 ± 0.011	44.71 ± 0.012
OM	92.98 ± 0.350	92.76 ± 0.302	92.54 ± 0.419	92.34 ± 0.610
CP	14.29 ± 0.952	14.40 ± 0.934	14.51 ± 0.917	14.63 ± 0.902
ADF	19.73 ± 0.849	20.27 ± 0.709	20.82 ± 0.703	21.36 ± 0.835
NDF	39.35 ± 2.063	40.32 ± 1.997	41.33 ± 1.939	42.31 ± 1.894
aNDFom	38.14 ± 2.016	38.92 ± 1.826	39.73 ± 1.681	40.53 ± 1.605
uNDF	12.23 ± 0.410	11.95 ± 0.400	11.65 ± 0.487	11.36 ± 0.635
Starch	24.10 ± 0.962	23.08 ± 0.983	22.03 ± 1.012	21.01 ± 1.049
Ether extract	3.33 ± 0.960	3.30 ± 0.104	3.26 ± 0.130	3.23 ± 0.165
Ca	0.54 ± 0.086	0.55 ± 0.088	0.57 ± 0.091	0.58 ± 0.094
P	0.31 ± 0.008	0.33 ± 0.005	0.35 ± 0.003	0.37 ± 0.003
Particle size distribution^4^, %				
> 19.0 mm	13.90 ± 2.590	14.56 ± 3.230	15.25 ± 4.196	15.91 ± 5.274
< 19.0 > 8.0 mm	39.48 ± 1.368	39.90 ± 1.814	40.34 ± 2.757	40.77 ± 3.815
< 8.0 > 4.0 mm	26.37 ± 4.742	25.53 ± 4.741	24.67 ± 4.748	23.83 ± 4.761
< 4.0 mm	20.26 ± 6.167	20.01 ± 6.055	19.75 ± 5.942	19.50 ± 5.833

^1^The control diet (BCON) contained 38.61% barley grain along with 59.62% barley silage (harvested at the soft dough stage of maturity) and 1.78% of a mineral, vitamin, and feed additive. Hybrid rye silage (harvested at the boot stage of maturity) replaced 0%, 33%, 67%, and 100% of the barley silage resulting in inclusions of hybrid rye silage of 19.67% (RLOW), 39.94% (RMED), and 59.62% (RHIGH) of dietary DM.

^2^The mineral supplement was obtained from Canadian Feed Research Center (North Battleford, SK, Canada) and contained: 15.08% of calcium, 0.63% of phosphorus, 1.02% of magnesium, 0.80% of potassium, 2.02% of sodium, 3.09% of chlorine, 0.18% of sulfur, 669.8 mg/kg of manganese, 311.3 mg/kg of copper, 1298.6 mg/kg of iron, 967.2 mg/kg of zinc, 40.2 mg/kg of iodine, 0.05 mg/kg of cobalt, 6.5 mg/kg of selenium, 126.2 KIU/kg of vitamin A, 15.0 KIU/kg of vitamin D_3_, 3.3 KIU/kg of vitamin E. The final supplement contained 161.5 mg/kg of monesin (Elance Animal Health, Greenfield, IN) on a DM basis.

^3^Chemical composition and physical analysis are expressed as means ± SD (*n* = 4). DM, dry matter; OM, organic matter; CP, crude protein; ADF, acid detergent fiber; NDF, Neutral detergent fiber; aNDFom, NDF measured using α-amylase, sodium sulfite, and corrected for ash content; uNDF, undigested NDF.

^4^Particle size distribution (*n* = 4) was calculated on an as-fed basis as described by Heinrichs (2013).

### DMI, ruminal pH, and ruminal fermentation

Increasing the HRS inclusion rate linearly decreased DMI, whether reported in kg/d or as a percentage of BW (*P* ≤ 0.04; Table 3). Water intake consumed from drinking linearly increased with increasing HRS inclusion (*P* = 0.02), while dietary water intake linearly decreased as HRS inclusion increased (*P* < 0.01). Total water intake (L/d) did not differ with increased HRS inclusion (*P* = 0.89).

**Table 3.** Effect of feeding hybrid rye silage as a replacement for barley silage on DMI, water intake, ruminal fermentation, and particle sorting of growing beef heifers

Item	Hybrid rye silage inclusion rate^1^				SEM^2^	*P*-value		
	BCON	RLOW	RMED	RHIGH		Treatment	Linear	Quadratic
Initial BW, kg	564	560	563	563	16.5	0.99	0.86	0.79
Final BW, kg	598	597	591	586	17.1	0.94	0.55	0.89
DMI, kg/d	10.14	10.22	9.70	9.22	0.420	0.15	0.04	0.40
DMI, % of BW	1.75	1.77	1.69	1.60	0.065	0.07	0.02	0.24
Total water intake^3^	33.2	35.9	34.7	33.8	2.04	0.50	0.89	0.18
Water intake (liquid), kg/d	18.6	21.7	21.9	22.5	1.69	0.06	0.02	0.25
Water intake (feed), kg/d	14.6	14.1	12.8	11.3	0.52	<0.01	<0.01	0.24
Particle sorting^4^, %								
> 19.0 mm	96.44	101.20	100.88	99.66	2.391	0.21	0.23	0.09
< 19.0 > 8.0 mm	95.82^a^	97.64	96.68^a^	95.21	1.650	0.47	0.59	0.16
< 8.0 > 4.0 mm	105.01^a^	102.88^a^	104.17^a^	105.89^a^	1.454	0.24	0.41	0.08
< 4.0 mm	103.50	99.44	100.74	102.69	2.750	0.19	0.86	0.04

^1^The control diet (BCON) contained 38.61% barley grain along with 59.62% barley silage (harvested at the soft dough stage of maturity) and 1.78% of a mineral, vitamin, and feed additive. Hybrid rye silage (harvested at the boot stage of maturity) replaced 0%, 33%, 67%, and 100% of the barley silage, resulting in inclusions of hybrid rye silage of 19.67% (RLOW), 39.94% (RMED), and 59.62% (RHIGH) of dietary DM.

^2^Greatest SEM was reported.

^3^Water intake was measured as feed water and water meter readings, respectively. Water density is assumed to be 1 kg/L.

^4^Particle sorting (*n* = 4) was calculated as (the actual intake of each fraction/the predicted intake of each fraction) × 100% on an as-fed basis as described by Leonardi and Armentano (2003).

^a^Indicates means that differ from 100% analyzed using a two-tailed t-test for each treatment.

Sorting index values for particles retained on the 19-mm sieve tended to be affected quadratically, with values increasing and then decreasing such that heifers fed CON sorted against long particles while those fed HRS did not sort for or against; however, none of the sorting index values for the 19-mm sieve differed from 100%. Sorting for particles retained on the 8-mm sieve were not affected by treatment. While heifers in all treatments preferentially consumed particles on the 4-mm sieve, the sorting index tended to respond quadratically (*P* = 0.08), where sorting for 4-mm particles decreased from BCON to RLOW and then increased with increasing HRS inclusion rate. In addition, a quadratic response was observed for the sorting index for particles retained on the pan, such that heifers fed BCON preferentially consumed these particles while those fed RLOW and RMED had lesser sorting index values, indicating no preferential consumption, and those fed RHIGH again preferentially consumed particles retained on the pan. No sorting index values for the pan differed from 100%.

Increasing the HRS inclusion as a replacement of BARS linearly increased the minimum (*P* < 0.01) and mean ruminal pH (*P* < 0.01), but did not affect maximum pH (Table 4). There was also a linear decrease for the duration that ruminal pH was <5.8 (*P* = 0.04). There was no diet effect on the area where ruminal pH was <5.8. Increasing HRS inclusion at the expense of BARS had no effect on total SCFA concentration (*P* = 0.46). However, the molar proportion of acetate linearly increased (*P* < 0.01) while propionate and butyrate linearly decreased (*P* < 0.01) as HRS inclusion increased as a replacement for BARS. Isobutyrate and isovalerate were not affected by dietary treatments, but the molar proportion of valerate linearly increased (*P* < 0.01) with increasing HRS inclusion. In addition, the ruminal NH_3_-N concentration linearly increased with increasing HRS inclusion in the diet (*P* = 0.03).

**Table 4.** Effect of feeding hybrid rye silage as a replacement for barley silage on ruminal fermentation of growing beef heifers^1^

Item	Hybrid rye silage inclusion rate^1^				SEM^2^	*P*-value		
	BCON	RLOW	RMED	RHIGH		Treatment	Linear	Quadratic
Ruminal pH								
Minimum pH	5.77	5.81	5.97	6.18	0.097	<0.01	<0.01	0.26
Mean pH	6.36	6.44	6.53	6.54	0.051	0.02	<0.01	0.42
Maximum pH	6.88	6.91	6.98	6.90	0.069	0.72	0.63	0.36
Duration < 5.8, min/d	94.5	59.1	8.4	4.2	34.66	0.19	0.04	0.64
Area < 5.8, (pH × min)/d	23.7	7.1	0.8	1.3	10.63	0.36	0.11	0.41
Rumen fermentation								
Total SCFA^3^, mM	105.6	106.5	105.6	104.2	2.84	0.78	0.46	0.47
SCFA proportions, mol/100 mol								
Acetate	62.11	63.57	64.07	64.93	0.526	<0.01	<0.01	0.52
Propionate	18.74	17.84	17.68	17.41	0.371	0.04	<0.01	0.47
Butyrate	13.94	13.46	12.76	12.34	0.409	0.03	<0.01	0.94
Isobutyrate	1.08	1.06	1.10	1.12	0.026	0.24	0.10	0.39
Isolvalerate	1.71	1.51	1.66	1.43	0.101	0.19	0.13	0.89
Valerate	1.72	1.73	1.88	1.95	0.053	<0.01	<0.01	0.57
NH_3_-N, mg/dL	8.75	9.03	9.61	10.24	0.581	0.16	0.03	0.72

^1^The control diet (BCON) contained 38.61% barley grain along with 59.62% barley silage (harvested at the soft dough stage of maturity) and 1.78% of a mineral, vitamin, and feed additive. Hybrid rye silage (harvested at the boot stage of maturity) replaced 0%, 33%, 67%, and 100% of the barley silage resulting in inclusions of hybrid rye silage of 19.67% (RLOW), 39.94% (RMED), and 59.62% (RHIGH) of dietary DM.

^2^Greatest SEM was reported.

^3^SCFA, short-chain fatty acids.

### Nutrient intake and the site and extent of digestion

Increasing HRS inclusion linearly decreased OM, uNDF, starch, and ether extract intake (*P* ≤ 0.02; Table 5) while ADF and aNDFom intake did not differ (*P* > 0.51). Increasing HRS inclusion also tended to decrease CP intake (*P* = 0.08). Consequently, the omasal flow of DM, OM, and aNDFom decreased linearly (*P* ≤ 0.01) as HRS replaced BARS. The apparent ruminal degradability of DM and OM responded quadratically, such that the values increased from BCON to RLOW and reached a plateau (*P* ≤ 0.02). The apparent ruminal aNDFom degradability increased linearly (*P *= 0.03) with increasing HRS inclusion. No difference was detected in ruminal starch degradability (*P* = 0.58).

**Table 5.** Effect of feeding hybrid rye silage as a replacement of barley silage on nutrient intake, digesta flow to omasum, and ruminal apparent degradability of nutrients of growing beef heifers^1^

Item	Hybrid rye silage inclusion rate^1^				SEM^2^	*P*-value		
	BCON	RLOW	RMED	RHIGH		Treatment	Linear	Quadratic
Nutrient intake^3^, kg/d								
OM	9.45	9.51	9.00	8.53	0.387	0.11	0.02	0.39
CP	1.45	1.47	1.41	1.35	0.059	0.26	0.08	0.37
ADF	1.98	2.07	2.02	1.94	0.089	0.69	0.65	0.29
aNDFom	3.88	4.04	3.90	3.74	0.192	0.73	0.51	0.40
aNDFom, % BW	0.67	0.68	0.68	0.65	0.027	0.49	0.50	0.17
uNDF	1.22	1.24	1.13	1.03	0.053	<0.01	<0.01	0.15
Starch	2.47	2.34	2.12	1.99	0.123	<0.01	<0.01	0.98
Ether extract	0.34	0.33	0.31	0.29	0.014	0.05	<0.01	0.43
Digesta flow to omasum, kg/d								
DM	7.88	7.12	6.77	6.56	0.363	0.03	<0.01	0.38
OM	5.98	5.23	4.97	4.78	0.291	<0.01	<0.01	0.25
aNDFom	2.47	2.10	1.94	1.94	0.175	0.05	0.01	0.23
Starch	0.08	0.10	0.06	0.10	0.019	0.29	0.88	0.53
Ruminal degradability, %								
DM	22.34	30.39	30.02	28.85	1.926	0.02	0.03	0.02
OM	36.72	45.24	44.79	44.08	1.930	<0.01	0.01	0.01
aNDFom	35.96	48.31	50.18	47.94	4.313	0.04	0.03	0.06
Starch	95.39	96.99	95.73	96.39	0.787	0.43	0.58	0.52

^1^The control diet (BCON) contained 38.61% barley grain along with 59.62% barley silage (harvested at the soft dough stage of maturity) and 1.78% of a mineral, vitamin, and feed additive. Hybrid rye silage (harvested at the boot stage of maturity) replaced 0%, 33%, 67%, and 100% of the barley silage, resulting in inclusions of hybrid rye silage of 19.67% (RLOW), 39.94% (RMED), and 59.62% (RHIGH) of dietary DM.

^2^Greatest SEM was reported.

^3^OM, organic matter; CP, crude protein; ADF, acid detergent fiber; aNDFom, neutral detergent fiber measured using α-amylase, sodium sulfite, and corrected for ash content; uNDF, undigested neutral detergent fiber.

Increasing HRS inclusion decreased intestinal digestibility (% of intake) of DM and OM in a quadratic manner (*P* ≤ 0.03; Table 6) such that digestibility decreased from BCON to RLOW and increased from RMED to RHIGH. There were no differences for the intestinal digestibility (% of intake) of aNDFom or starch (*P* > 0.10). When reported as a percentage of nutrient flow, increasing HRS inclusion resulted in a tendency for a quadratic effect on OM digestibility (*P* = 0.07), with RLOW and RMED tending to have lesser digestibility than BCON and RHIGH. There was no dietary effect on intestinal digestibility of DM, aNDFom, or starch (*P* > 0.10).

**Table 6.** Effect of feeding hybrid rye silage as a replacement for barley silage on apparent intestinal digestibility of nutrients of growing beef heifers

Item	Hybrid rye silage inclusion rate^1^				SEM^2^	*P*-value		
	BCON	RLOW	RMED	RHIGH		Treatment	Linear	Quadratic
Intestinal digestibility^3^, % of intake								
DM	41.53	35.88	35.97	38.92	1.872	0.11	0.34	0.03
OM	30.82	23.98	23.88	26.21	1.928	0.04	0.09	0.02
aNDFom	14.07	7.42	5.99	12.28	4.306	0.40	0.69	0.11
Starch	3.15	1.68	2.75	2.94	0.885	0.60	0.91	0.31
Intestinal digestibility, % of digesta flow to omasum								
DM	53.34	51.47	51.47	54.35	1.496	0.39	0.63	0.11
OM	48.37	43.79	43.20	46.30	2.135	0.26	0.45	0.07
aNDFom	18.30	13.17	10.73	21.03	5.961	0.52	0.81	0.17
Starch	54.20	43.98	55.39	61.49	10.828	0.69	0.46	0.42

^1^The control diet (BCON) contained 38.61% barley grain along with 59.62% barley silage (harvested at the soft dough stage of maturity) and 1.78% of a mineral, vitamin, and feed additive. Hybrid rye silage (harvested at the boot stage of maturity) replaced 0%, 33%, 67%, and 100% of the barley silage, resulting in inclusions of hybrid rye silage of 19.67% (RLOW), 39.94% (RMED), and 59.62% (RHIGH) of dietary DM.

^2^Greatest SEM was reported.

^3^DM, dry matter; OM, organic matter; NDF, Neutral detergent fiber; aNDFom, neutral detergent fiber measured using α-amylase, sodium sulfite, and corrected for ash content.

Increasing HRS inclusion linearly increased apparent total tract DM, ADF, and aNDFom digestibility (*P* < 0.01; Table 7), and tended to increase OM digestibility (*P* = 0.09). Total tract digestibility of CP, starch, and ether extract were not affected by dietary treatment (*P* > 0.10). Apparent total tract GE digestibility and dietary DE concentration did not differ. Fecal DM output linearly decreased with increasing HRS inclusion (*P* < 0.01).

**Table 7.** Effect of feeding hybrid rye silage as a replacement of barley silage on total tract apparent digestibility of nutrients, DE, and fecal output of growing beef heifers

Item	Hybrid rye silage inclusion rate^1^				SEM^2^	*P*-value		
	BCON	RLOW	RMED	RHIGH		Treatment	Linear	Quadratic
Digestibility^3^, % of intake								
DM	63.87	66.24	65.99	67.77	1.030	0.04	<0.01	0.74
OM	67.54	69.19	68.67	70.28	1.058	0.28	0.09	0.99
CP	64.50	66.17	65.04	65.45	1.255	0.72	0.71	0.54
ADF	34.49	42.87	44.48	49.13	2.005	<0.01	<0.01	0.33
aNDFom	50.03	55.57	56.17	60.22	1.502	<0.01	<0.01	0.61
Starch	98.18	98.28	98.03	98.37	0.273	0.77	0.77	0.63
Ether extract	64.17	66.53	67.06	68.02	2.262	0.58	0.19	0.74
GE	64.57	66.45	65.55	67.15	1.127	0.34	0.15	0.89
DE, Mcal/kg	2.82	2.90	2.87	2.93	0.053	0.40	0.17	0.86
Fecal output, kg/day	3.65	3.46	3.28	2.96	0.149	<0.01	<0.01	0.62

^1^The control diet (BCON) contained 38.61% barley grain along with 59.62% barley silage (harvested at the soft dough stage of maturity) and 1.78% of a mineral, vitamin, and feed additive. Hybrid rye silage (harvested at the boot stage of maturity) replaced 0%, 33%, 67%, and 100% of the barley silage, resulting in inclusions of hybrid rye silage of 19.67% (RLOW), 39.94% (RMED), and 59.62% (RHIGH) of dietary DM.

^2^Greatest SEM was reported.

^3^DM, dry matter; OM, organic matter; CP, crude protein; ADF, acid detergent fiber; aNDFom, neutral detergent fiber measured using α-amylase, sodium sulfite, and corrected for ash content; GE, gross energy; DE, digestible energy.

## Discussion

In the present study, a linear reduction in DMI (kg/day and % of BW) was observed as HRS inclusion increased when used as a replacement for BARS in diets for growing heifers. This aligns with our previous study with growing steers in which HRS was harvested at the late milk stage and replaced BARS harvested at the soft dough stage of maturity (Zhang et al., 2025). The reasons for reduced DMI with increasing HRS inclusion remain unclear; although Zhang et al. (2025) suggested that the increase in dietary NDF concentration with increasing HRS inclusion may lead to greater ruminal fill, or that awns present as part of the spikelet may reduce the palatability of rye. In the present study, aNDFom intake was less than 0.7% of BW, suggesting that it is unlikely that aNDFom intake should limit DMI (Mertens, 1987). Moreover, uNDF intake linearly decreased with increasing HRS inclusion and ruminal aNDFom degradability linearly increased, challenging whether increased aNDFom concentration is a plausible explanation driving reduced DMI.

Several authors have suggested that rye forage has low palatability, particularly as maturity advances (Lardy et al., 2022; O’Reilly, 2024), but we were not able to identify specific factors in the literature that may alter palatability. It should also be noted that as HRS inclusion increased, the dietary DM concentration increased from 40.55% for BCON to 44.71% for RHIGH. Most studies evaluating TMR DM concentration within the observed range for dietary DM in the present study have reported reductions in DMI as dietary DM decreases (Lahr et al., 1983; Miller-Cushon and DeVries, 2009; Felton and DeVries, 2010). Thus, the available data also challenge whether dietary DM explains the reduction in DMI as HRS replaces BARS. However, those studies evaluated the use of water inclusion rather than moisture inherent within a feed ingredient and as such, responses may differ.

The stage of maturity at the time of harvest can affect ensiling characteristics (Kim et al., 2001) and potentially palatability. It has been suggested that fall rye should be harvested at the flag leaf to flowering stage for silage (Kim et al., 2001) and hay production because of the potential decrease in palatability and intake (Stefanyshyn-Cote, 1993; Schlegel, 2013; McLelland and Brook, 2016), and reduction in IVDMD (Kim et al., 2001) with advancing maturity. Stefanyshyn-Cote (1993) suggested that fall rye harvested either as hay or as silage at flowering and mid-milk might display inherent factors that reduce intake when compared to barley silage harvested at the mid-dough stage, but did not describe what these potential factors could be. Awns are one possible inherent anti-palatability factor and are present at the boot stage. When comparing awn structure among cereals, rye awns contain a more lignified, thick-walled cell compared to barley and wheat (Evert, 2006; Ntakirutimana and Xie, 2019), which may influence the palatability of rye when harvested as whole-crop silage. However, the HRS in the present study was harvested at the boot stage, chopped, and ensiled, questioning whether awns may be a plausible factor driving the reduction in DMI. Future research is needed to elucidate factors that affect the palatability of HRS and to determine if this changes with the stage of maturity.

The decrease in DMI and reduction in dietary starch with increasing HRS inclusion were likely the primary contributing factors for many of the observed results in the present study. For example, the increased mean ruminal pH and the reduction in the duration that pH was <5.8 with increasing HRS inclusion could be caused by the reduction in DMI and the lesser dietary starch as HRS inclusion increased. Past studies that purposely imposed low feed intake reported that ruminal pH increases in a dose-dependent manner as feed intake decreases (Zhang et al., 2013), and more recently, a negative correlation between DMI and ruminal pH has been reported (Hartinger et al., 2024). The linear increase for the molar proportion of acetate and linear decrease for the molar proportions of propionate and butyrate are characteristic of diets with greater aNDFom, particularly considering the linear increase in ruminal degradability of aNDFom with increasing HRS inclusion, lesser dietary starch (Russell, 2002; NASEM, 2016; Chibisa et al., 2020), and when cattle decrease DMI (Zhang et al., 2013). Moreover, the reduction in fecal DM output was likely driven by reduced DMI.

Increasing inclusion of HRS linearly reduced aNDFom intake but linearly increased ruminal degradability and total tract digestibility of aNDFom. The earlier stage of maturity at harvest for HRS than for BARS likely explains the greater aNDFom digestibility. As noted, the HRS used in the present study was harvested at the boot stage of maturity as recommended by Heuzé et al. (2015) and O’Reilly (2024). When compared to BARS harvested at soft dough stage, HRS contained greater aNDFom but lesser uNDF than BARS, suggesting a greater supply of potentially degradable aNDFom supporting the digestibility responses observed. Kim et al. (2001) reported that the NDF concentration increased and IVDMD decreased for rye from the boot to flowering stages. Likewise, for barley forage, increasing maturity from late milk to hard dough decreased NDF digestibility (Rosser et al., 2016). However, few studies have compared digestibility responses between HRS and BARS when harvested at industry-recommended stages of maturity. In addition to differences in maturity, the reduction in DMI with increasing HRS inclusion may have helped facilitate greater aNDFom digestibility as decreased DMI is expected to extend ruminal retention, thereby enhancing ruminal DM and NDF degradability (Allen and Mertens, 1988; Russell et al., 1992; Allen, 2020).

The observed linear increase in ruminal NH_3_-N concentrations was likely a result of reduced fermentable substrate supply limiting energy availability for ruminal microorganisms to synthesize microbial protein (Russell et al., 1992; Russell, 2002; NASEM, 2016) and is consistent with the reduction in dietary starch and OM intake as HRS inclusion increased. Differences in maturity at the time of harvest between the HRS and BARS may help explain this response. Rye (Kim et al., 2001) and barley (Rosser et al., 2013) forage decrease in CP concentration with advancing maturity (Boudon et al., 2002). As such, it is likely that the reduction in dietary starch, coupled with greater CP with increasing HRS inclusion, led to the greater ruminal ammonia concentration observed. As we did not measure true ruminal CP degradability or urinary N output, the lack of response for apparent total tract CP digestibility is difficult to interpret. Future studies should include the evaluation of N-balance and whether HRS alters microbial protein production.

It is surprising that GE digestibility and dietary DE concentrations did not differ with increasing HRS inclusion rate, particularly when considering previous results evaluating the replacement of BARS with HRS for growing steers (Zhang et al., 2025). The lack of response for GE and DE may be associated with the reduced DMI (Allen, 1996) and improved ruminal degradability of DM, OM, and aNDFom, as well as apparent total tract digestibility of DM, ADF, and aNDFom, with increasing HRS inclusion. Thus, in the present study, the increase in DM, OM, and aNDFom digestibility likely compensated for the decreased starch concentration, resulting in similar GE digestibility and DE concentration amongst treatments.

There are some limitations in the present study. Firstly, forages were harvested at industry-recommended maturities for ensiling (Stefanyshyn-Cote, 1993; Nair et al., 2016) rather than at the same stage of maturity. Maturity at harvest impacts quality and the nutritional value of whole-plant forages along with the resulting silage (Borreani et al., 2018; Nair et al., 2018; Moore et al., 2020). For example, the HRS had greater DM content than BARS. The BARS DM concentration was less than ideal, which could impact ensiling characteristics, challenging whether the lesser DM concentration of the BARS could help explain the reduction in DMI observed with increasing HRS inclusion. However, based on silage pH, organic acid concentrations, and ammonia, both silages appeared to be adequately preserved. Additionally, small grain species are known to have differing digestibility at the same stage of maturity (Stefanyshyn-Cote, 1993), and the changes for chemical composition (e.g., CP and soluble CP, aNDFom, and starch) and digestibility differ by plant species with advancing maturity (Helsel and Thomas, 1987; Stefanyshyn-Cote, 1993; Edmisten et al., 1998b). While the in vitro and in situ degradability of NDF decreases as barley (Rosser et al., 2013; Nair et al., 2018) and fall rye (Helsel and Thomas, 1987; Stefanyshyn-Cote, 1993; Kantar et al., 2011) advance in maturity, the digestibility of rye has been reported to be lesser than barley at all stages of maturity evaluated (Helsel and Thomas, 1987).

Another limitation is that we used a simple ingredient substitution approach for dietary formulation, such that HRS replaced BARS. The approach used resulted in greater dietary DM and NDF, and lesser dietary starch as HRS inclusion increased. While some nutritionists may prioritize the proportion of forage in the growing diet, findings from this study and a related study (Zhang et al., 2025) suggest that dietary formulation strategies should also consider the dietary aNDFom and starch concentrations. It is unclear whether responses for DMI, ruminal fermentation, and nutrient digestibility would be similar if diets were formulated for equal starch and aNDFom. However, ingredient substitution studies are a logical first step when evaluating novel ingredients (Swanson et al., 2017).

## Conclusion

Replacing BARS with increasing levels of HRS in growing diets decreased DMI and increased ruminal pH without affecting total SCFA concentration in the rumen. The increased extent of ruminal DM, OM, and aNDFom degradability with increasing HRS inclusion can be interpreted to indicate that harvesting HRS at an earlier stage of maturity than BARS may stimulate ruminal NDF degradability, although the reduction in DMI may have also contributed to this response. Incorporating more HRS in diets increased the ruminal NH_3_-N concentration, which might imply lower efficiency of ruminal fermentation and microbial protein synthesis; however, the latter was not assessed. Given the lack of difference for apparent total tract GE digestibility and the resulting diet DE concentration, there is likely a tradeoff between the greater digestibility of aNDFom in HRS and less starch than in BARS. In conclusion, the use of HRS, harvested at the boot stage, as a direct replacement for BARS, harvested at the soft dough stage, is likely to reduce DMI but improve NDF digestibility without affecting dietary DE concentration.

## Abbreviations:

ADF: acid detergent fiber
aNDFom: ash-free neutral detergent fiber
BW: body weight
CP: crude protein
DE: digestible energy
DM: dry matter
DMI: dry matter intake
BCON: the control diet with 59.62% BARS included for heifers
GE: gross energy
NDF: neutral detergent fiber
NEg: net energy for gain
NEm: net energy for maintenance
OM: organic matter
RHIGH: the growing diet with 100% of BARS replaced by HRS
RLOW: the growing diet with 33% of BARS replaced by HRS
RMED: the growing diet with 67% of BARS replaced by HRS
RUP: ruminally undegradable protein
SCFA: short-chain fatty acid
uNDF: undigested neutral detergent fiber

## References

[CIT0001] Allen, M. S. 1996. Physical constraints on voluntary intake of forages by ruminants. J. Anim. Sci. 74:3063–3075. doi: https://doi.org/10.2527/1996.74123063x 8994921

[CIT0002] Allen, M. S. 2020. Review: control of feed intake by hepatic oxidation in ruminant animals: integration of homeostasis and homeorhesis. Animal 14:s55–s64. doi: https://doi.org/10.1017/S1751731119003215 32024573

[CIT0003] Allen, M. S., and D. R. Mertens. 1988. Evaluating constraints on fiber digestion by rumen microbes. J. Nutr. 118:261–270. doi: https://doi.org/10.1093/jn/118.2.261 2828582

[CIT0004] Ankom Technology Corp. 2017. Detergent fiber in feeds - filter gag technique (for A200 and A200I). Macedon, NY, USA. [accessed on January 26, 2025] https://www.ankom.com/sites/default/files/document-files/Method_6_NDF_A200.pdf?srsltid=AfmBOooohBynSpFZv1f5jSYrFidWJMkWbXwDhiRzg6_aUpiUdvtOyss1

[CIT0005] Beauchemin, K. A., W. Z. Yang, and L. M. Rode. 2001. Effects of barley grain processing on the site and extent of digestion of beef feedlot finishing diets. J. Anim. Sci. 79:1925–1936. doi: https://doi.org/10.2527/2001.7971925x 11465381

[CIT0007] Borreani, G., E. Tabacco, R. J. Schmidt, B. J. Holmes, and R. E. Muck. 2018. Silage review: Factors affecting dry matter and quality losses in silages. J. Dairy Sci. 101:3952–3979. doi: https://doi.org/10.3168/jds.2017-13837 29685272

[CIT0006] Boudon, A., J. -L. Peyraud, and P. Faverdin. 2002. The release of cell contents of fresh rye-grass (*Lolium perenne* L.) during digestion in dairy cows: effect of the intracellular constituents, season and stage of maturity. Anim. Feed Sci. Technol. 97:83–102. doi: https://doi.org/10.1016/s0377-8401(01)00350-9

[CIT0008] Brito, A. F., G. F. Tremblay, H. Lapierre, A. Bertrand, Y. Castonguay, G. Bélanger, R. Michaud, C. Benchaar, D. R. Ouellet, and R. Berthiaume. 2009. Alfalfa cut at sundown and harvested as baleage increases bacterial protein synthesis in late-lactation dairy cows. J. Dairy Sci. 92:1092–1107. doi: https://doi.org/10.3168/jds.2008-1469 19233802

[CIT0009] Broderick, G. A., and J. H. Kang. 1980. Automated simultaneous determination of ammonia and total amino acids in ruminal fluid and in vitro media. J. Dairy Sci. 63:64–75. doi: https://doi.org/10.3168/jds.S0022-0302(80)82888-8 7372898

[CIT0011] Chibisa, G. E., D. A. Christensen, and T. Mutsvangwa. 2012. Effects of replacing canola meal as the major protein source with wheat dried distillers grains with solubles on ruminal function, microbial protein synthesis, omasal flow, and milk production in cows. J. Dairy Sci. 95:824–841. doi: https://doi.org/10.3168/jds.2011-4718 22281346

[CIT0010] Chibisa, G. E., K. A. Beauchemin, K. M. Koenig, and G. B. Penner. 2020. Optimum roughage proportion in barley-based feedlot cattle diets: total tract nutrient digestibility, rumination, ruminal acidosis, short-chain fatty absorption, and gastrointestinal tract barrier function. J. Anim. Sci. 98:skaa160. doi: https://doi.org/10.1093/jas/skaa160 32447367 PMC7447917

[CIT0013] Darambazar, E., K. Larson, D. Damiran, and H. A. Lardner. 2022. Evaluation of new fall rye cultivar ‘Bono’ in single and double cropping systems. Agronomy 12:1382. doi: https://doi.org/10.3390/agronomy12061382

[CIT0014] Edmisten, K. L., J. T. Green, J. P. Mueller, and J. C. Burns. 1998a. Winter annual small grain forage potential. I. Dry matter yield in relation to morphological characteristics of four small grain species at six growth stages. Commun. Soil Sci. Plant Anal. 29:867–879. doi: https://doi.org/10.1080/00103629809369992

[CIT0015] Edmisten, K. L., J. T. Green, J. P. Mueller, and J. C. Burns. 1998b. Winter annual small grain forage potential. II. Quantification of nutritive characteristics of four small grain species at six growth stages. Commun. Soil Sci. Plant Anal. 29:881–899. doi: https://doi.org/10.1080/00103629809369993

[CIT0016] Evert, R. 2006. Sclerenchyma. In: Esau’s plant anatomy, meristems, cells, and tissues of the plant body: their structure, function, and development. 3rd ed. Hoboken, New Jersey, USA: John Wiley & Sons, Inc; p. 191–209

[CIT0017] Felton, C. A., and T. J. DeVries. 2010. Effect of water addition to a total mixed ration on feed temperature, feed intake, sorting behavior, and milk production of dairy cows. J. Dairy Sci. 93:2651–2660. doi: https://doi.org/10.3168/jds.2009-3009 20494174

[CIT0018] Fisher, L. J., and J. R. Lessard. 1987. Intake and digestibility of corn, rye and sorghum-sudan grass silages by lactating cows. Can. J. Anim. Sci. 67:1027–1032. doi: https://doi.org/10.4141/cjas87-108

[CIT0019] France, J., and R. C. Siddons. 1986. Determination of digesta flow by continuous market infusion. J. Theor. Biol. 121:105–119. doi: https://doi.org/10.1016/s0022-5193(86)80031-5

[CIT0020] Geiger, H. H., and T. Miedaner. 2009. Rye (*Secale cereale L*.). In: M. J. Carena, editor. Cereals. Handbook of plant breeding, vol. 3. New York, NY: Springer Science and Business Media; p. 157–181.

[CIT0021] Hartinger, T., E. Castillo-Lopez, N. Reisinger, and Q. Zebeli. 2024. Elucidating the factors and consequences of the severity of rumen acidosis in first-lactation Holstein cows during transition and early lactation. J. Anim. Sci. 102:skae041. doi: https://doi.org/10.1093/jas/skae041 38364366 PMC10946224

[CIT0022] Heinrichs, J. 2013. The Penn State particle separator. University Park, PA: Penn State Extension. [accessed on October 17, 2024]. https://extension.psu.edu/penn-state-particle-separator

[CIT0023] Helsel, Z. R., and J. W. Thomas. 1987. Small grains for forage. J. Dairy Sci. 70:2330–2338. doi: https://doi.org/10.3168/jds.s0022-0302(87)80293-x

[CIT0024] Heuzé V. , Tran G., and P. Nozière. 2015. Rye forage. Feedipedia, a programme by INRAE, CIRAD, AFZ and FAO. [accessed on October 22, 2024]. https://www.feedipedia.org/node/385

[CIT0026] Johnson, J. A., B. D. Sutherland, J. J. McKinnon, T. A. McAllister, and G. B. Penner. 2020. Use of barley or corn silage when fed with barley, corn, or a blend of barley and corn on growth performance, nutrient utilization, and carcass characteristics of finishing beef cattle. . Transl Anim Sci 4:129–140. doi: https://doi.org/10.1093/tas/txz168 32704973 PMC6994044

[CIT0028] Kantar, M., Sheaffer, C., Porter, P., Krueger, E., and Ochsner, T. E. 2011. Growth stage influences forage yield and quality of winter rye. Online. Forage and Grazinglands. doi: https://doi.org/10.1094/FG-2011-0126-01-RS

[CIT0029] Khorasani, G. R., E. K. Okine, and J. J. Kennelly. 1996. Forage source alters nutrient supply to the intestine without influencing milk yield. J. Dairy Sci. 79:862–872. doi: https://doi.org/10.3168/jds.S0022-0302(96)76435-4 8792286

[CIT0030] Kim, J. G., E. S. Chung, S. Seo, J. S. Ham, W. S. Kang, and D. A. Kim. 2001. Effects of maturity at harvest and wilting days on quality of round baled silage. Asian-Aust. J. Anim. Sci. 14:1233–1237. doi: https://doi.org/10.5713/ajas.2001.1233

[CIT0031] KWS Production Guide Hybrid Rye. 2023. KWS Cereal USA, LLC. Champaign, IL. [accessed on October 16, 2024]. https://www.kws.com/us/en/resources/crop-management/hybrid-rye-downloads/

[CIT0027] Lahr, D. A., D. E. Otterby, D. G. Johnson, J. G. Linn, and R. G. Lundquist. 1983. Effects of moisture content of complete diets on feed intake and milk production by cows. J. Dairy Sci. 66:1891–1900. doi: https://doi.org/10.3168/jds.S0022-0302(83)82027-X 6630672

[CIT0032] Lardy, G., V. Anderson, C. Dahlen, and Z. Carlson. 2022. Alternative feeds for ruminants. North Dakota State University Extension Publication AS1182, Fargo, North Dakota, USA. [accessed on September 13, 2024]. https://www.ndsu.edu/agriculture/extension/publications/alternative-feeds-ruminants

[CIT0033] Leonardi, C., and L. E. Armentano. 2003. Effect of quantity, quality, and length of alfalfa hay on selective consumption by dairy cows. J. Dairy Sci. 86:557–564. doi: https://doi.org/10.3168/jds.S0022-0302(03)73634-0 12647962

[CIT0035] McLelland, M., and H. Brook. 2016. Fall rye production. Alberta Agriculture and Forestry, Edmonton, AB, Canada.

[CIT0036] Mertens, D. R. 1987. Predicting intake and digestibility using mathematical models of ruminal function. J. Anim. Sci. 64:1548–1558. doi: https://doi.org/10.2527/jas1987.6451548x 3583960

[CIT0037] Miller-Cushon, E. K., and T. J. DeVries. 2009. Effect of dietary dry matter concentration on the sorting behavior of lactating dairy cows fed a total mixed ration. J. Dairy Sci. 92:3292–3298. doi: https://doi.org/10.3168/jds.2008-1772 19528606

[CIT0039] Moore, K. J., A. W. Lenssen, and S. L. Fales. 2020. Factors affecting forage quality. In: Forages. John Wiley & Sons, Ltd. p. 701–717. https://onlinelibrary.wiley.com/doi/abs/10.1002/9781119436669.ch39. [accessed on December 17, 2024]

[CIT0041] Nair, J., D. Christensen, P. Yu, A. D. Beattie, T. McAllister, D. Damiran, N. Preston, L. Fuhr, and J. J. McKinnon. 2016. A nutritional evaluation of common barley varieties grown for silage by beef and dairy producers in western Canada. Can. J. Anim. Sci. 96:598–608. doi: https://doi.org/10.1139/cjas-2016-0032

[CIT0042] Nair, J., D. Christensen, P. Yu, T. McAllister, N. Preston, D. Damiran, and J. J. McKinnon. 2017. Effect of variety and level of inclusion of barley varieties for silage selected to vary in neutral detergent fiber digestibility on performance and carcass characteristics of growing and finishing beef steers. Can. J. Anim. Sci. 97:383–394. doi: https://doi.org/10.1139/cjas-2016-0109

[CIT0040] Nair, J., A. D. Beattie, D. Christensen, P. Yu, T. McAllister, D. Damiran, and J. J. McKinnon. 2018. Effect of variety and stage of maturity at harvest on nutrient and neutral detergent fiber digestibility of forage barley grown in western Canada. Can. J. Anim. Sci. 98:299–310. doi: https://doi.org/10.1139/cjas-2017-0060

[CIT0043] National Academies of Sciences, Engineering, and Medicine (NASEM). 2016. Nutrient requirements of beef cattle. 8th rev. ed. Washington, DC: The National Academies Press. doi:doi: https://doi.org/10.17226/19014

[CIT0044] Ntakirutimana, F., and W. Xie. 2019. Morphological and genetic mechanisms underlying awn development in monocotyledonous grasses. Genes (Basel) 10:573. doi: https://doi.org/10.3390/genes10080573 31366144 PMC6723108

[CIT0045] O’Reilly, C. 2024. Guide to forage production. Publication 30. Ontario Ministry of Agriculture, Food and Rural Affairs. Ontario, Canada. https://www.ontario.ca/page/publication-30-guide-forage-production. [accessed on October 22, 2024]

[CIT0046] Penner, G. B., K. A. Beauchemin, and T. Mutsvangwa. 2006. An evaluation of the accuracy and precision of a stand-alone submersible continuous ruminal pH measurement system. J. Dairy Sci. 89:2132–2140. doi: https://doi.org/10.3168/jds.S0022-0302(06)72284-6 16702280

[CIT0047] Penner, G. B., J. A. Johnson, B. D. Sutherland, L. P. Clark, and C. J. Elford. 2020. Effects of drinking water sulfate concentrations on feed and water intake, growth, and serum mineral concentrations in growing beef heifers. Appl. Anim. Sci. 36:201–207. doi: https://doi.org/10.15232/aas.2019-01919

[CIT0049] Rosser, C. L., P. Górka, A. D. Beattie, H. C. Block, J. J. McKinnon, H. A. Lardner, and G. B. Penner. 2013. Effect of maturity at harvest on yield, chemical composition, and in situ degradability for annual cereals used for swath grazing. J. Anim. Sci. 91:3815–3826. doi: https://doi.org/10.2527/jas.2012-5677 23658356

[CIT0048] Rosser, C. L., A. D. Beattie, H. C. Block, J. J. McKinnon, H. A. Lardner, P. Górka, and G. B. Penner. 2016. Effect of maturity at harvest for whole-crop barley and oat on dry matter intake, sorting, and digestibility when fed to beef cattle. J. Anim. Sci. 94:697–708. doi: https://doi.org/10.2527/jas.2015-0063 27065140

[CIT0050] Russell, J. B. 2002. Rumen Microbiology and Its Role in Ruminant Nutrition. Department of Microbiology, Cornell University.

[CIT0051] Russell, J. B., J. D. O’Connor, D. G. Fox, P. J. Van Soest, and C. J. Sniffen. 1992. A net carbohydrate and protein system for evaluating cattle diets: I. Ruminal fermentation. J. Anim. Sci. 70:3551–3561. doi: https://doi.org/10.2527/1992.70113551x 1459918

[CIT0052] Sartin, A., K. Calus, A. Hall, M. Grabau, D. Redfearn, and M. E. Drewnoski. 2022. Effect of species and maturity on winter hardy small grain silage yield and quality. J. Anim. Sci. 100:1–2. doi: https://doi.org/10.1093/jas/skac313.001

[CIT0053] Schlegel, R. H. J. 2013. Rye: Genetics, Breeding, and Cultivation. CRC Press, Boca Raton.

[CIT0054] Siddons, R. C., J. Paradine, D. E. Beever, and P. R. Cornell. 1985. Ytterbium acetate as a particulate-phase digesta-flow marker. Br. J. Nutr. 54:509–519. doi: https://doi.org/10.1079/bjn19850136 2998454

[CIT0056] Stefanyshyn-Cote, Barbara Ann. 1993. The effect of maturity and ensiling on the nutritional quality of fall rye (*Secale cereale L*.) forage. University of Saskatchewan, Saskatoon, SK, Canada.

[CIT0057] Swanson, K. C., Z. E. Carlson, M. C. Ruch, T. C. Gilbery, S. R. Underdahl, F. E. Keomanivong, M. L. Bauer, and A. Islas. 2017. Influence of forage source and forage inclusion level on growth performance, feeding behavior, and carcass characteristics in finishing steers. J. Anim. Sci. 95:1325–1334. doi: https://doi.org/10.2527/jas.2016.1157 28380528

[CIT0058] Terler, G., T. Gruber, T. Hartinger, and Q. Zebeli. 2025. Effects of replacing rye silage with mixed rye-vetch-straw on feed intake, milk production, digestion processes, and blood metabolites in dairy cows. J. Dairy Sci. 108:5942–5953. doi: https://doi.org/10.3168/jds.2024-26060 40222676

[CIT0059] Udén, P., P. E. Colucci, and P. J. Van Soest. 1980. Investigation of chromium, cerium and cobalt as markers in digesta. Rate of passage studies. J. Sci. Food Agric. 31:625–632. doi: https://doi.org/10.1002/jsfa.2740310702 6779056

[CIT0060] Yang, W. Z., K. A. Beauchemin, and L. M. Rode. 2000. Effects of barley grain processing on extent of digestion and milk production of lactating cows. J. Dairy Sci. 83:554–568. doi: https://doi.org/10.3168/jds.S0022-0302(00)74915-0 10750114

[CIT0063] Zhang, S., R. I. Albornoz, J. R. Aschenbach, D. R. Barreda, and G. B. Penner. 2013. Short-term feed restriction impairs the absorptive function of the reticulo-rumen and total tract barrier function in beef cattle. J. Anim. Sci. 91:1685–1695. doi: https://doi.org/10.2527/jas.2012-5669 23422009

[CIT0062] Zhang, F., R. E. Carey, R. S. Brattain, H. Wehrle, and G. B. Penner. 2024. Effects of feeding hybrid rye grain as a replacement for barley grain on dry matter intake, ruminal fermentation, and the site and extent of nutrient digestion in finishing beef heifers. J. Anim. Sci. 102:skae211. doi: https://doi.org/10.1093/jas/skae211 39051129 PMC11329801

[CIT0061] Zhang, F., R. S. Brattain, H. Wehrle, V. Baron, G. O. Ribeiro, and G. B. Penner. 2025. Yield of hybrid rye silage and its use as a replacement for barley silage on feed intake, growth performance, and carcass quality of growing and finishing steers. Transl. Anim. Sci. 9:txaf048. doi: https://doi.org/10.1093/tas/txaf048 40336823 PMC12057561

